# Serotonin in the Frontal Cortex: A Potential Therapeutic Target for Neurological Disorders

**DOI:** 10.4172/2167-0501.1000e184

**Published:** 2016-12-29

**Authors:** Hui Lu, Qing-song Liu

**Affiliations:** 1 Department of Pharmacology and Physiology, George Washington University, Washington, DC 20037, USA; 2 Department of Pharmacology and Toxicology, Medical College of Wisconsin, Milwaukee, W 53226, USA

Serotonin or 5-hydroxytryptamine (5-HT) is a monoamine neurotransmitter which has broad distribution in the brain. It was discovered by Erspamer and Asero in the 1950s [1]. 5-HT is synthesized in two steps, with Tryptophan Hydroxylase (TPH) as the rate-limiting enzyme [2]. First, tryptophan is converted to 5-hydroxytryptophan (5-HTP) by TPH. Second, the intermediate product, 5-HTP, is converted to 5-HT by aromatic acid decarboxylase (AADC). 5-HT is primarily degraded by the mitochondrial bound protein Monoamine Oxidase A (MAOA), leading to the generation of the metabolite, 5-hydroxyindoleacetic acid (5-HIAA). Importantly, serotonin is also a substrate for melatonin synthesis [3]. 5-HT is released from the axonal terminals of serotoninergic neurons and acts on 14 distinct receptor subtypes that are classified into 7 different families: 5-HT1 (1A, 1B, 1D, 1E, 1F), 5-HT2 (2A, 2B, and 2C), 5-HT3, 5-HT4, 5-HT5 (5A, 5B), 5-HT6, and 5-HT7. Among all these receptors, only 5-HT3 receptor is a pentameric ligand-gated ion channel composed of several subunits of which 5 different types have been identified [4]. All other 5-HT receptors are G-protein coupled receptors which regulate the activity of the neurons expressing them [5,6]. Released serotonin is transported to the presynaptic neurons by serotonin transporter (SERT or 5-HTT), a type of monoamine transporter protein [7].

Serotonergic neurons are located in the raphe nuclei [8]. While the more caudal raphe nuclei project to the Peripheral Nervous System (PNS), the neurons in the dorsal and median raphe nuclei (DRN and MRN) primarily send their projections to forebrain regions [9,10]. 5-HT is critically involved in the development of many cortices, such as somatosensory cortex and barrel cortex [11,12]. In adult brain, 5-HT neurons project to majority of cortical areas, including the entorhinal and cingulate cortices. However, of all cortical regions, the frontal lobe contains the highest density of serotonergic terminals and 5-HT receptors [13]. These studies indicate that 5-HT regulates cognitive and emotional functions that rely on frontal cortical activity.

Serotonin homeostasis in the frontal cortex is important for normal behavior. It has been shown that engaging in aggressive behavior triggers dynamic changes in frontal cortical serotonin [14]. Deviation of 5-HT homeostasis increases impulsivity [15–17]. Selective depletion of 5-HT in monkey frontal cortex impairs reversal leaning and increases perseveration (loss of cognitive flexibility) [18,19]. 5-HT in the frontal cortex also modulates attention in humans [20,21]. Given its involvement in cognition and impulsivity, 5-HT is central to our understanding of the psychopathology and treatment of psychiatric disorders such as depression, schizophrenia, Obsessive Compulsive Disorder (OCD), and autism. In all of these disorders, local abnormalities in frontal cortex structure [22], neurochemistry [23], or activation [24] have been characterized and drugs for these disorders, such as ecstasy [25] and amphetamine [26], have been shown to impair cortical 5-HT neurotransmission. Moreover, Clarke et al. [18] used a serial discrimination reversal paradigm to show that selective depletion of 5-HT in the marmoset frontal cortex produced perseverative responding to the stimulus previously paired with reward without any significant effects on either retention of a discrimination learned preoperatively or acquisition of a novel discrimination postoperatively. This result highlights the importance of prefrontal serotonin in behavioral flexibility which is highly relevant to obsessive-compulsive disorder, schizophrenia, and the cognitive sequelae of drug abuse in which perseveration is prominent. More interestingly, 5-HT is very likely to be the common neurochemical factor between depression and autism as comorbidity of these two disorders is common [27]. Indeed, selective serotonin reuptake inhibitors (SSRIs) are being increasingly used in autism, because of their role in the control of depression and aggression [27–29].

Furthermore, high-frequency electrical stimulation of the Ventral Medial Prefrontal Cortex (vmPFC) is capable of enhancing 5-HT release and restoring social approach behaviour in defeated mice [30]. Additionally, manipulating vmPFC synaptic inputs to the DRN has revealed bidirectional effects on socioaffective behaviours via direct monosynaptic excitation and indirect disynaptic inhibition of 5-HT neurons [31]. Similarly, deep brain stimulation in the vmPFC improves negative bias and symptoms of mood dysregulation in Major Depressive Disorder (MDD) patients [32]. Strikingly, these results suggest that cross-species parallels exist in regards to the roles of the frontal cortex and serotonergic systems in socioaffective responses. It has been hypothesized that the plasticity of the frontal cortex-DRN circuit that links these two systems may constitute a conserved means of encoding or expressing social avoidance behaviour across species [31]. Thus, better understanding the interaction between frontal cortex and DRN and identifying key neuroplastic events that mediate normal and pathological regulation of socioaffective functions may uncover molecular targets amenable to therapeutic intervention in the treatment of affective disorders.
